# The Influence of *BMX* Gene Polymorphisms on Clinical Symptoms after Mild Traumatic Brain Injury

**DOI:** 10.1155/2014/293687

**Published:** 2014-04-22

**Authors:** Yu-Jia Wang, Yu-Wen Hsu, Che-Mai Chang, Chung-Che Wu, Ju-Chi Ou, Yan-Rou Tsai, Wen-Ta Chiu, Wei-Chiao Chang, Yung-Hsiao Chiang, Kai-Yun Chen

**Affiliations:** ^1^Graduate Institute of Neural Regenerative Medicine, College of Medical Science and Technology, Taipei Medical University, Taipei 11031, Taiwan; ^2^Department of Pharmacy, Wan Fang Hospital, Taipei Medical University, Taipei 11696, Taiwan; ^3^Department of Clinical Pharmacy, School of Pharmacy, Taipei Medical University, Taipei 11031, Taiwan; ^4^Department of Neurosurgery, Taipei Medical University Hospital, Taipei 11031, Taiwan; ^5^Department of Emergency Medicine, Shuang-Ho Hospital, Taipei Medical University, New Taipei City 23561, Taiwan; ^6^Ministry of Health and Welfare, Taipei 10341, Taiwan; ^7^Master Program for Clinical Pharmacogenomics and Pharmacoproteomics, School of Pharmacy, Taipei Medical University, Taipei 11031, Taiwan; ^8^Center for Neurotrauma and Neuroregeneration, Taipei Medical University, Taipei 11031, Taiwan

## Abstract

Mild traumatic brain injury (mTBI) is one of the most common neurological disorders. Most patients diagnosed with mTBI could fully recover, but 15% of patients suffer from persistent symptoms. In recent studies, genetic factors were found to be associated with recovery and clinical outcomes after TBI. In addition, results from our previous research have demonstrated that the bone marrow tyrosine kinase gene in chromosome X *(BMX)*, a member of the Tec family of kinases, is highly expressed in rats with TBI. Therefore, our aim in this study was to identify the association between genetic polymorphisms of *BMX* and clinical symptoms following mTBI. Four tagging single nucleotide polymorphisms (tSNPs) of *BMX* with minimum allele frequency (MAF) >1% were selected from the HapMap Han Chinese database. Among these polymorphisms, rs16979956 was found to be associated with the Beck anxiety inventory (BAI) and dizziness handicap inventory (DHI) scores within the first week after head injury. Additionally, another SNP, rs35697037, showed a significant correlation with dizziness symptoms. These findings suggested that polymorphisms of the *BMX* gene could be a potential predictor of clinical symptoms following mTBI.

## 1. Introduction 

Traumatic brain injury (TBI) is an important cause of mortality and disability worldwide. TBI is usually caused by a blunt or penetrating trauma or an external force to the head, leading to damage of the normal function of the brain [1]. The neurobiological damage following TBI is a complex mechanism, which causes long-term physical, cognitive, and emotional impairment, resulting in enormous medical and social expenses. TBI can be classified as mild, moderate, and severe according to Glasgow Coma Scale (GCS) categories [2]. Among these three classifications, mild TBI (mTBI) constitutes most instances of TBI each year [3]. Most patients diagnosed with mTBI recover fully within days to months, but up to 15% of patients will continue to experience persistent functional or emotional symptoms [4, 5]. To discover reliable genetic biomarkers for recovery from TBI, recent studies have focused on associations between genetic factors and neuroscience [6–8].

Previous genetic association studies have indicated that genetic susceptibility is related to recovery and clinical outcomes following TBI. Genetic variations of the apolipoprotein E (*APOE*) gene have been demonstrated to be associated with long-term functional outcome after TBI [9–11]. Polymorphisms of brain-derived neurotrophic factor (*BDNF*), monoamine oxidase A (*MAO-A*), and interleukin-6 (IL-6) have also been correlated with TBI in recent studies [12–14]. Results of these studies identified several candidate genes associated with TBI, but these results are still controversial and need further validation. There may still be other genetic polymorphisms that correlate with the pathogenesis of TBI.

A member of the Tec family of kinases, the bone marrow tyrosine kinase gene in chromosome X (*BMX*), which is also known as epithelial/endothelial tyrosine kinase (*ETK*) [15], plays an important role in immune functions and regulation via its involvement in intracellular signaling mechanisms of cytokine receptors, lymphocyte surface antigens, heterotrimeric G-protein coupled receptors, and integrin molecules. Most literature, thus far, regards* BMX* as a modulator of apoptosis and cancer cell growth, and its cell-specific function has been characterized in various cancer cells [16, 17]. Studies have also shown the BMX-dependent pathway to be crucial in ischemic brain injury for the recruitment of inflammatory cells and angiogenesis at the site of injury [18–20]. Genetic profiling suggests that cytokine-mediated recruitment of inflammatory cells is involved in BMX-induced chronic inflammation and angiogenesis [21]. In addition, BMX was reported to regulate Toll-like receptor-induced IL-6 production, a cytokine closely related to traumatic brain injury [22–24]. Our recent study using an animal model revealed that BMX is expressed at the injured site and correlates with various levels of TBI, suggesting the potential for BMX as a neurotrauma biomarker [25].

To obtain comprehensive insight into the function of* BMX* in TBI, our aim in this study was to identify the association of* BMX* genetic polymorphisms with clinical symptoms following mTBI. Since emotional function represents a prominent domain of impairment after mTBI, the relationship between the SNPs of* BMX* and posttraumatic anxiety and depression symptoms was investigated. Additionally, since dizziness is an important symptom of postconcussion syndrome following mTBI, we also assessed the association of* BMX *polymorphisms with dizziness of patients.

## 2. Materials and Methods

### 2.1. Patients Studied

Data were collected at three hospitals associated with Taipei Medical University. An independent researcher identified patients with mTBI, injured between September 2010 and December 2012. All patients completed evaluations by a well-trained nurse at baseline and at a 6th week follow-up visit. This study protocol was approved by the TMU-Joint Institutional Review Board.

### 2.2. *BMX* ELISA Assay

Whole blood samples were collected from mTBI patients and healthy volunteers after informed consent, and the sera were stored at −80°C until testing. Sera were assessed for the antibody against cytoplasmic tyrosine-protein kinase (anti-BMX) using a commercial specific enzyme-linked immunosorbent assay (ELISA) kit (USCN, Wuhan, China), with a range of 1.56–100 *μ*g/L, according to the manufacturer's instruction. Results were determined by measuring optical density of each well, using a microplate reader Infinite 200 (TECAN, Morrisville, NC) with wavelength set to 450 nm. A standard curve was created by reducing the data using computer software Magellan (TECAN, Morrisville, NC) capable of generating a four-parameter logistic (4-PL) curve-fit and calculating the sample concentrations.

### 2.3. Self-Reported Questionnaires

The Beck anxiety inventory (BAI) is a 21-item self-reported measure of subjective, somatic, or panic-related symptoms of anxiety [26]. A higher score represents a higher level of anxiety (range 0–63). Cut-off ranges are as follows: 0–9 (no anxiety), 10–18 (mild to moderate), 19–29 (moderate to severe), and 30–63 (severe anxiety). The BAI has been used in several studies of TBI patients [27–29].

The Beck depression inventory (BDI) is a 21-item self-reported measure of cognitive, behavioral, and physiological symptoms associated with depression [30]. Subjects are asked to choose the item that best describes how they have been feeling in the past week, with higher scores reflecting a greater severity of depression symptoms (range 0–63). Cut off ranges are as follows: 0–9 (minimal depressive symptoms), 10–18 (mild depression), 19–29 (moderate depression), and 30–63 (severe depression).

The dizziness handicap inventory (DHI) is a 25-item self-reported questionnaire, with a total score between 0 and 100. Higher scores indicate a greater level of dizziness in daily life. The 25 items describe three aspects: functional problems (9 items), emotional problems (9 items), and physical problems (7 items). The test-retest reliability and internal consistency of the DHI are excellent [31].

### 2.4. DNA Extraction and Genotyping

DNA was extracted from blood cells by an initial treatment with 0.5% sodium dodecyl sulfate lysis buffer, followed by proteinase K (1 mg/mL) to digest nuclear proteins, for 4 h at 60°C. Total DNA was harvested using a Gentra (Qiagen, Inc., Valencia, CA) extraction kit followed by 70% alcohol precipitation. Genotyping for four SNPs was carried out using the TaqMan Allelic Discrimination Assay (Applied Biosystems, Foster City, CA). A polymerase chain reaction (PCR) used a 96-well microplate with the ABI9700 Thermal Cycler (Applied Biosystems, Foster City, CA). After the PCR, StepOne software, version 2.2.2 (Applied Biosystems, Foster City, CA), was used to detect and analyze the fluorescence.

### 2.5. Statistical Analysis

JMP 9.0 for Windows was used for all statistical analyses. Student's* t*-test was used to compare the mean of continuous variables (Age, DHI score, BDI score, and BMX serum level) between mTBI patients and control subjects, as well as different* BMX* genotypes. Statistical differences between the nominal variables (gender, headache, depression, dizziness, severity of BAI, and BDI) were evaluated by a *χ* ^2^-test with one degree of freedom. Fisher's exact test was performed when 20% of cells had expected counts of less than five. A *P* value less than 0.05 was considered significant.

## 3. Results 

### 3.1. Differential Serum* BMX* Level and Clinical Features between mTBI Patients and Controls

**Figure 1:**
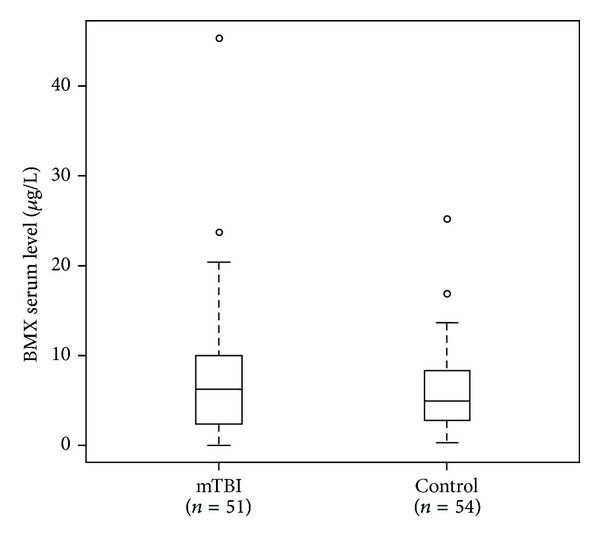
Differential serum BMX level between mTBI patients and controls.

Initially, we compared serum BMX levels in a total of 51 mTBI patients and 54 controls. The mean serum level of BMX was higher in mTBI patients (7.47 *μ*g/L) than in healthy controls (6.08 *μ*g/L) (Figure 1). The characteristics of these subjects are shown in Table 1. The mean patient age was 42.3 in mTBI patients and 30.9 in the controls. The percentage of females was different between mTBI patients and control groups (66.7% and 59.3%, resp.). Our data revealed that 27.5% of the mTBI patients suffered from headache, which was slightly higher than controls (20.4%). Of note, the mTBI patients had a higher depression symptoms rating (54.9%) than control subjects (16.7%), and the result was consistent with BDI scores. In addition, mTBI patients had higher DHI scores, which reflected the effects of dizziness on their quality of life.

### 3.2. Selection of tSNPs of* BMX* for the Association Study with mTBI

**Figure 2:**
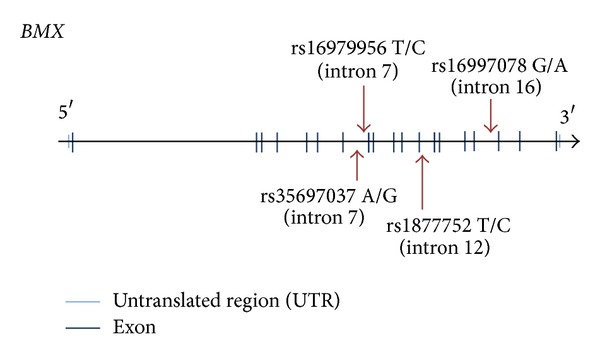
The graphical overview of the genotyped polymorphisms identified in relation to the exon/intron structure of* BMX* gene.

In order to further define the association between the* BMX* gene and clinical symptoms in mTBI patients, we enlarged the patient sample size to investigate the correlation of genetic variants with mTBI. A total of 217 mTBI patients were recruited, ranging from 18 to 83 years, with a mean age of 38.5. Of the recruited patients, 62.2% (135/217) were female (Table 2). Four tagging SNPs in* BMX* (rs35697037, rs16979956, rs1877752, and rs16997078) with a minimum allele frequency (MAF) of >1% were selected from the HapMap Han Chinese database (http://www.hapmap.org/). All of these SNPs were located in the* BMX* intronic region (Figure 2).

### 3.3. Association between Beck Inventory Score and Genetic Polymorphisms of BMX

We examined whether the* BMX* SNPs were associated with the Beck anxiety inventory (BAI) and Beck depression inventory (BDI) scores. These questionnaires were completed one and six weeks after head injury. We classified the scores into four grades and compared the lower grades (Grade 1 and Grade 2) and the higher grades (Grade 3 and Grade 4) between the different genotypes of* BMX*. Patients carrying the T allele (TT and CT genotypes) of rs16979956 showed higher grades of BAI during the first week than patients who carried the CC genotype (*P* value = 0.0117, OR, 95% CI = 12.08, 1.06–138.01) (Table 3(a)), but the significance was not confirmed by Fisher's exact test. The overall BAI scores decreased at the sixth week, but none of these SNPs revealed a significant correlation with anxiety symptoms (Table 3(b)). We also calculated the BDI score, another clinical outcome of mTBI. However, we failed to find an association between* BMX* and BDI score at the first week (Table 4(a)) and sixth week (Table 4(b)) after head injury.

### 3.4. Association between Dizziness and Genetic Polymorphisms of* BMX*

**Figure 3:**
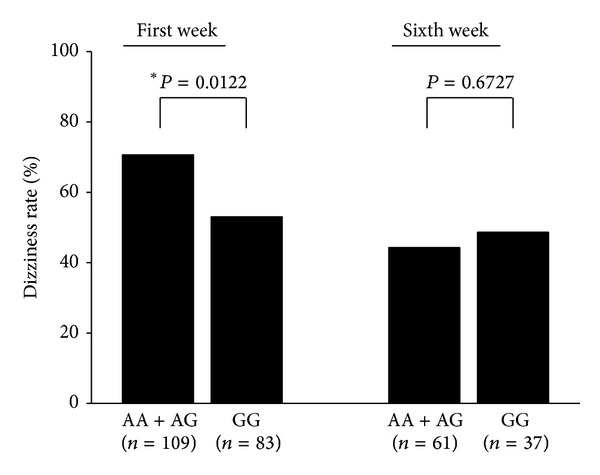
Dizziness rate among mTBI patients stratified by rs35697037.

We further analyzed the association of* BMX* SNPs and symptoms in mTBI patients. As shown in Table 5, mTBI patients with TT and CT genotypes (57.0 ± 46.7) of rs16979956 had higher dizziness handicap inventory (DHI) scores than patients with the CC genotype (25.7 ± 21.5) during the first week (*P* value = 0.0445). In addition, we found another SNP (rs35697037) at the same intronic region that also had a propensity to affect the DHI score (*P* value = 0.0738). However, we found no significant association between* BMX* polymorphisms and DHI score after six weeks. To validate this result, we examined the dizziness rate from nurses' recordings. As shown in Figure 3, the AA and AG genotypes (70.6%) of SNP (rs35697037) carriers were significantly associated with a higher incidence of dizziness than GG (53.0%) genotype carriers within the first week (*P* value = 0.0122). However, we failed to find a significant correlation between the other SNPs of* BMX *and the occurrence rate of dizziness (data not shown).

## 4. Discussion

Previous genetic association studies have supported the notion that genetic susceptibility can affect traumatic injury recovery. The association between genetic polymorphisms and TBI has been investigated in recent studies, but there is still no widely accepted biomarker.

Our previous data have shown that the expression of BMX is 1.8- to 2.5-fold higher in rats with induced traumatic brain injury than in naïve rats. By using quantitative PCR and western blot analysis, we also demonstrated that BMX is increasingly elevated in mTBI rats in a time-dependent manner. In addition, the level of BMX correlated with the severity of trauma [25]. In this study, we provide evidence that BMX in serum is higher in mTBI patients than in healthy controls, consistent with the findings from our mTBI animal model studies. Moreover, we found that* BMX* polymorphisms are associated with dizziness symptoms following mTBI.

Postconcussion symptoms (PCS), which can last from months to years, are the most common complaints following mTBI [32]. These symptoms include cognitive complaints (decreased memory and concentration), somatic complaints (tiredness, headache, dizziness, tinnitus, and insomnia), and affective complaints (depression, irritability, and anxiety) [33]. Predictors that allow early detection of patients who are at risk of developing PCS have not been clearly established. In recent studies, patients with dizziness after mTBI were found to have a higher risk of developing persistent PCS [34–36]. Early anxiety is also a potential predictor of persistent PCS [37]. Our results showed that patients carrying the T allele variant in rs16979956 (T/T and C/T genotypes) had higher DHI scores, which represents the impact of dizziness on quality of life. Patients with the T allele variant also had higher BAI scores, but the statistical significance disappeared after adjustment using Fisher's exact test. With another SNP in* BMX*, rs35697037, the A/A, and A/G genotypes also contributed to higher dizziness rates and had the propensity to affect the DHI score. The genetic polymorphisms of* BMX* may be early predictors of dizziness symptoms and persistent PCS.

Because rs16979956 and rs35697037 are located in the intron of the* BMX *gene, allelic differences are not expected to alter the amino acid sequence of BMX. However, the functional significance of* BMX* variations should be determined by follow-up studies.

To our knowledge, this is the first report showing that polymorphic variations in the* BMX* gene may be associated with clinical symptoms following mTBI. Our previous research based on an animal model and this genetic association study both support the potential of BMX as a predictor of TBI.

## Figures and Tables

**Table 1:** Clinical characteristics of patients with mTBI and healthy individuals as controls.

	mTBI	Control
Sample size	51	54
Age	42.33 ± 15.75	30.85 ± 7.9*
Gender (Female)	34 (66.67%)	32 (59.26%)
Headache (%)	14 (27.45%)	11 (20.37%)
Depression (%)	28 (54.90%)	9 (16.67%)*
DHI	30.35 ± 23.47	6.51 ± 14.58*
BDI	10.04 ± 9.04	4.55 ± 5.42*
Injury type		
Car accident	21	—
Falls	20	—
Others	10	—

*P value < 0.05.

**Table 2:** Basal characteristics of patients with mild traumatic brain injury (mTBI).

Characteristics	Patients with mTBI
Number of subjects	217
Gender: female, no. (%)	135 (62.2)
Age (years)^a^	38.5 ± 14.6
Range	18–83
GCS^a^	15.0 ± 0.2
GOSE^a^	6.7 ± 1.1
Injury type	
Accident	191 (88.0)
Attacked	23 (10.6)
Unknown	3 (1.4)

^a^Mean ± SD. SD: standard deviation.

**Table:** (a)

SNP	Genotype	Number	Grade 1 (0–9)	Grade 2 (10–18)	Grade 3 (19–29)	Grade 4 (30–63)	^ a^OR (95% CI) *P* value	^ b^Fisher's exact test *P* value
rs35697037	AA + AG	107	67 (62.6)	22 (20.6)	14 (13.1)	4 (3.7)	1.40	
	GG	79	50 (63.3)	19 (24.0)	7 (8.9)	3 (3.8)	(0.61–3.21)	
							0.5352	—

rs16979956	TT + CT	3	1 (33.3)	0 (0.0)	1 (33.3)	1 (33.3)	12.08	
	CC	183	116 (63.4)	41 (22.4)	20 (10.9)	6 (3.3)	(1.06–138.01)	
							0.0117*	0.0597

rs1877752	TT + CT	9	4 (44.4)	2 (22.2)	3 (33.3)	0 (0.0)	3.04	
	CC	177	113 (63.8)	39 (22.0)	18 (10.2)	7(4.0)	(0.71–12.95)	
							0.1159	0.1372

rs16997078	GG + AG	7	4 (57.1)	1 (14.3)	0 (0.0)	2 (28.6)	2.35	
	AA	179	113 (63.1)	40 (22.4)	21 (11.7)	5 (2.8)	(0.43–12.78)	
							0.3080	0.2841

*P < 0.05 is labeled in bold. ^a^Grade 1 + Grade 2 versus Grade 3 + Grade 4. ^b^20% of cells has expected count less than 5, Fisher's exact test would be performed.

**Table:** (b)

SNP	Genotype	Number	Grade 1 (0–9)	Grade 2 (10–18)	Grade 3 (19–29)	Grade 4 (30–63)	^ a^OR (95% CI) *P* value	^ b^Fisher's exact test * P* value
rs35697037	AA + AG	64	46 (71.9)	10 (15.6)	7 (10.9)	1 (1.6)	1.29	
	GG	40	32 (80.0)	4 (10.0)	3 (7.5)	1 (2.5)	(0.36–4.58)	
							0.6978	0.7635

rs16979956	TT + CT	1	1 (100.0)	0 (0.0)	0 (0.0)	0 (0.0)	0	
	CC	103	77 (74.8)	14 (13.6)	10 (9.7)	2 (1.9)	—	
							0.7167	1.0000

rs1877752	TT + CT	4	3 (75.0)	1 (25.0)	0 (0.0)	0 (0.0)	0	
	CC	100	75 (75.0)	13 (13.0)	10 (10.0)	2 (2.0)	—	
							0.4614	1.0000

rs16997078	GG + AG	4	2 (50.0)	1 (25.0)	1 (25.0)	0 (0.0)	2.70	
	AA	100	76 (76.0)	13 (13.0)	9 (9.0)	2 (2.0)	(0.26–28.23)	
							0.3901	0.3923

^a^Grade 1 + Grade 2 versus Grade 3 + Grade 4. ^b^20% of cells has expected count less than 5, Fisher's exact test would be performed.

**Table:** (a)

SNP	Genotype	Number	Grade 1 (0–9)	Grade 2 (10–18)	Grade 3 (19–29)	Grade 4 (30–63)	^ a^OR (95% CI) *P* value	^ b^Fisher's exact test * P* value
rs35697037	AA + AG	107	66 (61.7)	30 (28.0)	8 (7.5)	3 (2.8)	0.81	
	GG	81	53 (65.4)	18 (22.2)	8 (9.9)	2 (2.5)	(0.33–2.0)	
							0.6562	—

rs16979956	TT + CT	3	3 (100.0)	0 (0.0)	0 (0.0)	0 (0.0)	0	
	CC	185	116 (62.7)	48 (26.0)	16 (8.6)	5 (2.7)	—	
							0.5358	1.0000

rs1877752	TT + CT	9	7 (77.8)	1 (11.1)	1 (11.1)	0 (0.0)	0.99	
	CC	179	112 (62.6)	47 (26.2)	15 (8.4)	5 (2.8)	(0.12–8.36)	
							0.9954	1.0000

rs16997078	GG + AG	7	5 (71.4)	1 (14.3)	1 (14.3)	0 (0.0)	1.34	
	AA	181	114 (63.0)	47 (26.0)	15 (8.3)	5 (2.8)	(0.15–11.72)	
							0.7897	0.5698

^a^Grade 1 + Grade 2 versus Grade 3 + Grade 4. ^b^20% of cells has expected count less than 5, Fisher's exact test would be performed.

**Table:** (b)

SNP	Genotype	Number	Grade 1 (0–9)	Grade 2 (10–18)	Grade 3 (19–29)	Grade 4 (30–63)	^ a^OR (95% CI) *P* value	^ b^Fisher's exact test * P* value
rs35697037	AA+ AG	64	42 (65.6)	13 (20.3)	7 (11.0)	2 (3.1)	1.96	
	GG	39	29 (74.4)	7 (17.9)	3 (7.7)	0 (0.0)	(0.50–7.75)	
							0.3284	0.5280

rs16979956	TT+CT	1	0 (0.0)	1 (100.0)	0 (0.0)	0 (0.0)	0	
	CC	102	71 (69.6)	19 (18.6)	10 (9.8)	2 (2.0)	—	
							0.7152	1.0000

rs1877752	TT+CT	4	2 (50.0)	1 (25.0)	1 (25.0)	0 (0.0)	2.67	
	CC	99	69 (69.7)	19 (19.2)	9 (9.1)	2 (2.0)	(0.25–27.92)	
							0.3960	0.3955

rs16997078	GG + AG	4	3 (75.0)	0 (0.0)	1 (25.0)	0 (0.0)	2.67	
	AA	99	68 (68.7)	20 (20.2)	9 (9.1)	2 (2.0)	(0.25–27.92)	
							0.3960	0.3955

^a^Grade 1 + Grade 2 versus Grade 3 + Grade 4. ^b^20% of cells has expected count less than 5, Fisher's exact test would be performed.

**Table 5:** Difference in DHI score among mTBI patients stratified by different *BMX* genotypes.

SNP	Genotype	Number	First week^a^	Number	Sixth week^a^
rs35697037	AA + AG	110	28.5 ± 22.3	66	16.7 ± 21.7
	GG	80	22.7 ± 21.1	50	11.7 ± 16.0
	* P*-value		0.0738		0.1751

rs16979956	TT + CT	2	57.0 ± 46.7	2	2.0 ± 2.8
	CC	188	25.7 ± 21.5	114	14.8 ± 19.6
	*P*-value		0.0445*		0.3615

rs1877752	TT + CT	8	21.3 ± 17.4	5	9.6 ± 14.2
	CC	182	26.3 ± 22.1	111	14.8 ± 19.8
	*P*-value		0.5288		0.5645

rs16997078	GG + AG	7	29.7 ± 29.3	4	20.5 ± 18.1
	AA	183	25.9 ± 21.7	112	14.3 ± 19.6
	*P*-value		0.6534		0.5376

*P < 0.05 is labeled in bold. ^a^Mean ± SD. SD: standard deviation.

## References

[1] Coronado VG, McGuire LC, Sarmiento K (2012). Trends in Traumatic Brain Injury in the U.S. and the public health response: 1995–2009. *Journal of Safety Research*.

[2] Teasdale G, Jennett B (1974). Assessment of coma and impaired consciousness. A practical scale. *The Lancet*.

[3] Arciniegas DB, Anderson CA, Topkoff J (2005). Mild traumatic brain injury: a neuropsychiatric approach to diagnosis, evaluation, and treatment. *Neuropsychiatric Disease and Treatment*.

[4] Marshall S, Bayley M, McCullagh S, Velikonja D, Berrigan L (2012). Clinical practice guidelines for mild traumatic brain injury and persistent symptoms. *Canadian Family Physician*.

[5] Silver JM, McAllister TW, Arciniegas DB (2009). Depression and cognitive complaints following mild traumatic brain injury. *The American Journal of Psychiatry*.

[6] Lingsma HF, Roozenbeek B, Steyerberg EW, Murray GD, Maas AI (2010). Early prognosis in traumatic brain injury: from prophecies to predictions. *The Lancet Neurology*.

[7] Zetterberg H, Smith DH, Blennow K (2013). Biomarkers of mild traumatic brain injury in cerebrospinal fluid and blood. *Nature Reviews Neurology*.

[8] Dash PK, Zhao J, Hergenroeder G, Moore AN (2010). Biomarkers for the diagnosis, prognosis, and evaluation of treatment efficacy for traumatic brain injury. *Neurotherapeutics*.

[9] Teasdale GM, Nicoll JAR, Murray G, Fiddes M (1997). Association of apolipoprotein E polymorphism with outcome after head injury. *The Lancet*.

[10] Zhou W, Xu D, Peng X, Zhang Q, Jia J, Crutcher KA (2008). Meta-analysis of APOE4 allele and outcome after traumatic brain injury. *Journal of Neurotrauma*.

[11] Chiang M-F, Chang J-G, Hu C-J, Dunn L, Nicoll JAR (2003). Association between apolipoprotein E genotype and outcome of traumatic brain injury. *Acta Neurochirurgica*.

[12] Dardiotis E, Fountas KN, Dardioti M (2010). Genetic association studies in patients with traumatic brain injury. *Neurosurgical Focus*.

[13] Dardiotis E, Grigoriadis S, Hadjigeorgiou GM (2012). Genetic factors influencing outcome from neurotrauma. *Current Opinion in Psychiatry*.

[14] Weaver SM, Chau A, Portelli JN (2012). Genetic polymorphisms influence recovery from traumatic brain injury. *The Neuroscientist*.

[15] Robinson D, He F, Pretlow T, Kung H-J (1996). A tyrosine kinase profile of prostate carcinoma. *Proceedings of the National Academy of Sciences of the United States of America*.

[16] Chen K-Y, Huang L-M, Kung H-J, Ann DK, Shih H-M (2004). The role of tyrosine kinase Etk/Bmx in EGF-induced apoptosis of MDA-MB-468 breast cancer cells. *Oncogene*.

[17] Bagheri-Yarmand R, Mandal M, Taludker AH (2001). Etk/Bmx tyrosine kinase activates Pak1 and regulates tumorigenicity of breast cancer cells. *The Journal of Biological Chemistry*.

[18] Chen K-Y, Wu C-C, Chang C-F (2012). Suppression of Etk/Bmx protects against ischemic brain injury. *Cell Transplantation*.

[19] He Y, Luo Y, Tang S (2006). Critical function of Bmx/Etk in ischemia-mediated arteriogenesis and angiogenesis. *Journal of Clinical Investigation*.

[20] Jasielska M, Semkova I, Shi X (2010). Differential role of tumor necrosis factor (TNF)-*α* receptors in the development of choroidal neovascularization. *Investigative Ophthalmology and Visual Science*.

[21] Paavonen K, Ekman N, Wirzenius M, Rajantie I, Poutanen M, Alitalo K (2004). Bmx tyrosine kinase transgene induces skin hyperplasia, inflammatory angiogenesis, and accelerated wound healing. *Molecular Biology of the Cell*.

[22] Helmy A, Carpenter KLH, Skepper JN, Kirkpatrick PJ, Pickard JD, Hutchinson PJ (2009). Microdialysis of cytokines: methodological considerations, scanning electron microscopy, and determination of relative recovery. *Journal of Neurotrauma*.

[23] Palmer CD, Mutch BE, Workman S, McDaid JP, Horwood NJ, Foxwell BMJ (2008). Bmx tyrosine kinase regulates TLR4-induced IL-6 production in human macrophages independently of p38 MAPK and NF*κ*B activity. *Blood*.

[24] Dai B, Kim O, Xie Y (2006). Tyrosine kinase Etk/BMX is up-regulated in human prostate cancer and its overexpression induces prostate intraepithelial neoplasia in mouse. *Cancer Research*.

[25] Wu JC, Chen KY, Yu YW (2012). Location and level of Etk expression in neurons are associated with varied severity of traumatic brain injury. *PloS ONE*.

[26] Beck AT, Epstein N, Brown G, Steer RA (1988). An inventory for measuring clinical anxiety: psychometric properties. *Journal of Consulting and Clinical Psychology*.

[27] Wood R, Doughty C (2013). Alexithymia and avoidance coping following traumatic brain injury. *The Journal of Head Trauma Rehabilitation*.

[28] Wood RLL, Williams C (2008). Inability to empathize following traumatic brain injury. *Journal of the International Neuropsychological Society*.

[29] Bryant RA, Moulds M, Guthrie R, Nixon RDV (2003). Treating acute stress disorder following mild traumatic brain injury. *The American Journal of Psychiatry*.

[30] Beck AT, Ward CH, Mendelson M (1961). An inventory for measuring depression. *Archives of General Psychiatry*.

[31] Jacobson GP, Newman CW (1990). The development of the Dizziness Handicap Inventory. *Archives of Otolaryngology—Head and Neck Surgery*.

[32] Spinos P, Sakellaropoulos G, Georgiopoulos M (2010). Postconcussion syndrome after mild traumatic brain injury in Western Greece. *Journal of Trauma—Injury, Infection and Critical Care*.

[33] McAllister TW, Arciniegas D (2002). Evaluation and treatment of postconcussive symptoms. *NeuroRehabilitation*.

[34] Savola O, Hillbom M (2003). Early predictors of post-concussion symptoms in patients with mild head injury. *European Journal of Neurology*.

[35] Yang C-C, Hua M-S, Tu Y-K, Huang S-J (2009). Early clinical characteristics of patients with persistent post-concussion symptoms: a prospective study. *Brain Injury*.

[36] Chamelian L, Feinstein A (2004). Outcome after mild to moderate traumatic brain injury: the role of dizziness. *Archives of Physical Medicine and Rehabilitation*.

[37] Dischinger PC, Ryb GE, Kufera JA, Auman KM (2009). Early predictors of postconcussive syndrome in a population of trauma patients with mild traumatic brain injury. *Journal of Trauma—Injury, Infection and Critical Care*.

